# Germline *PDCDL1* Gene Variants Are Associated with Increased Primary Melanoma Thickness

**DOI:** 10.3390/biom15040584

**Published:** 2025-04-15

**Authors:** Elizabeth Córdoba-Lanús, Omar García-Pérez, Leticia Melgar-Vilaplana, Angélica Domínguez-de-Barros, Ricardo Fernández-de-Misa

**Affiliations:** 1 Instituto Universitario de Enfermedades Tropicales y Salud Pública de Canarias (IUETSPC), Universidad de La Laguna, Avda. Astrofísico Sánchez, s/n, 38296 San Cristóbal de La Laguna, Spain; ogarciap@ull.edu.es (O.G.-P.);; 2 Consorcio Centro de Investigación Biomédica (CIBER) de Enfermedades Infecciosas (CIBERINFEC), Instituto de Salud Carlos III, 28029 Madrid, Spain; 3 Pathology Department, Hospital Universitario Nuestra Señora de Candelaria, Ctra. Gral. del Rosario, 145, 38010 Santa Cruz de Tenerife, Spain; leticiamvfreedom@gmail.com; 4 Research Unit, Hospital Universitario Nuestra Señora de Candelaria, Ctra. Gral. del Rosario, 145, 38010 Santa Cruz de Tenerife, Spain; 5 Dermatology Department, Hospital Universitario Nuestra Señora de Candelaria, Ctra. Gral. del Rosario, 145, 38010 Santa Cruz de Tenerife, Spain; 6 Department of Internal Medicine, Dermatology and Psychiatry, Universidad de La Laguna, 38071 San Cristóbal de La Laguna, Spain

**Keywords:** *PDCDL1*, polymorphisms, melanoma

## Abstract

**Background**: The incidence of malignant melanoma (MM) continues to increase annually, and tumour invasiveness is a main prognostic factor. Single-nucleotide polymorphisms (SNPs) have become key tools in the study of cancer genetics, influencing susceptibility and prognosis. **Methods**: In the present study, we analysed the relationship between five SNPs on the *PDCDL1* gene (rs822336, rs822337, rs822338, rs229736, rs4143815) with prognosis as well as primary tumour invasiveness characteristics in 377 whole blood samples from MM individuals. **Results**: Patients who presented the rs822336 CG or GG genotypes (OR = 3.01, 95% CI = 1.53–5.92; *p* = 0.0017), TA or TT in rs822337 (OR = 2.45, 95% CI = 1.22–4.93; *p* = 0.0098), and CT or CC of rs822338 (OR = 2.23, 95% CI = 1.05–4.73; *p* = 0.028) were at an increased risk of developing invasive melanomas. Cases with the AG or GG genotype in rs2297136 presented a lower risk (OR = 0.29, 95% CI = 0.11–0.75; *p* = 0.0038) of invasive MM. The genetic analysis at the haplotype level resulted in similar findings (OR: 2.95, 95% CI: 1.08–8.10), *p* = 0.036). Furthermore, patients carrying the homozygous AA genotype in rs2297136 had thicker tumours than those harbouring the AG or GG (1.4 mm vs. 1.0 and 0.8 mm; *p* = 0.030). No significant association was found between the studied SNPs and melanoma-specific survival (MSS) nor progression-free survival (PFS). **Conclusions**: Current results suggest that SNPs rs822336, rs822337, rs822338, and rs2297136 genotypes in the *PDCDL1* gene are associated with the risk of tumour invasiveness and tumour thickness in MM. Further studies on SNPs considering genetic and epigenetic factors are needed for a better understanding of malignant melanoma susceptibility and its prognosis.

## 1. Introduction

The incidence of malignant melanoma (MM), both *in situ* and invasive, has increased significantly over the past 5 decades [1]. *In situ* MM is potentially curable with early surgery as the malignant cells do not spread beyond the epidermis. Spain accounts for 16% of MM [1,2]. When neoplastic cells invade the dermis or deeper layers, MM is considered invasive. Overall, the prognosis of invasive MM is favourable because more than half of the patients are diagnosed in the early stages of the disease [1]. However, the prognosis deteriorates significantly from stage II onward [3]. Breslow index (tumour thickness) and ulceration in the primary tumour are the prognostic factors for survival and define the T category in the AJCC 8th edition [3,4]. T category, together with nodal status (N) and visceral status (M), enables determining the clinical or pathological stage of the patient, thus establishing an estimation of the patient’s prognosis. As the stage increases, the survival of the patients worsens, shifting from a 5-year survival of 99% for stage IA patients to 32% for stage IIID patients [3].

So far, blood LDH levels are the only included serological prognostic biomarker in the AJCC 8th edition [5]. However, multiple prognostic biomarkers have been suggested [4]. Improved tests will likely be available soon for a more accurate, more individualized, and less aggressive estimation of the prognosis [6].

The presence of certain immune system regulators during the growth of various cancers deserves extensive studies. One of the most important immune system regulators is the axis PD1-PDL1. This interaction exerts an immune homeostasis, vital for developing the immune system and regulating peripheral and central tolerance [7]. This immune homeostasis can be altered in the tumour microenvironment due to mutations or signalling pathway activation (among others) that drive the overexpression of PD-L1 in tumour cells, preventing them from being recognised by the immune system [8]. The release of IFN-γ by T lymphocytes and NK cells, together with the JAK/STAT, MAPK, and NF-KB pathways, is the main precursor of PD-L1 activation [9,10]. PD-L1 is expressed on different tumour cells such as head and neck squamous cell carcinoma, MM, and carcinomas of the oesophagus, lung, breast, etc. [11]. Tumour cells take advantage of the immune system of individuals to express PD-L1 and generate an immunosuppressive tumour microenvironment. PD-L1 overexpression in the primary tumour confers a worse prognosis in multiple neoplasms [12,13].

In MM, there is limited and conflicting information on PD-L1 and its relationship with prognosis. The prevalence of PD-L1 expression in melanoma varies from 24% to 49%, although it has been seen in approximately 60% of tumours of chronically sun-damaged skin [14]. Furthermore, PD-L1 independently predicts a worse prognosis as it is correlated with tumour thickness and lymphatic and visceral dissemination [14]. On the other hand, there is evidence of favourable responses (prior to treatment) in MM without PDL1 expression [15]. This is why there is great controversy about the use and effectiveness of immunotherapeutic treatments based on PD-L1 expression in the tumour.

PD-L1 is encoded by the *PDCDL1* gene located on p24.1 of chromosome 9 [16] and consists of seven exons that encode a type 1 transmembrane protein of 40 kDa and 290 amino acids [14]. Various single-nucleotide polymorphisms (SNPs) have been identified in *PDCDL1* that increase susceptibility to developing various tumours, such as lung [17], gastric [18], or oesophageal cancer [19]. The development of high-throughput sequencing technologies made it easier to identify various SNPs in *PDCDL1*, leading to the discovery of new potential biomarkers for cancer. Some SNPs are specifically correlated with various tumour behaviours. It has been suggested that rs822336 and rs822337 are associated with unfavourable prognoses in triple-negative breast cancer patients [20]. A meta-analysis by Zou et al. [18], reported that the variant rs4143815 C > G increased the susceptibility to gastric and bladder cancers [18]. Furthermore, single-nucleotide polymorphisms in *PDCDL1* could be important regarding the response to treatment. Nomizo et al. [21] observed that individuals with advanced non-small-cell lung cancer carrying the rs4143815 CC genotype showed an improvement in the response to nivolumab [21]. Thus, in lung cancer, rs822336 [22], rs822337 [22], and rs4143815 [23] act as prognostic markers once the primary tumour has been removed. Another important *PDCDL1* SNP is rs2297136; it has been reported that patients carrying the genotype AG vs. GG were at increased risk of lung cancer [17]. In addition, patients with epithelial ovarian cancer carriers of the G allele of rs2297136 presented a shorter progression-free survival (PFS) than those patients harbouring the AA genotypes [24].

There is a lack of knowledge regarding the effect of SNPs present in the *PDCDL1* gene in patients with MM, as well as their potential association with the prognosis of the disease. The search for biomarkers has become a highly relevant aspect of personalising the prognosis of each MM patient and is crucial in the early stages of the disease. The present study aimed to define the associations between specific SNPs in the *PDCDL1* gene (rs822336, rs822337, rs822338, rs229736, rs4143815) and the main MM features and course. This would substantially improve the management of patients with melanoma.

## 2. Materials and Methods

### 2.1. Patients and Study Samples

**Figure 1:**
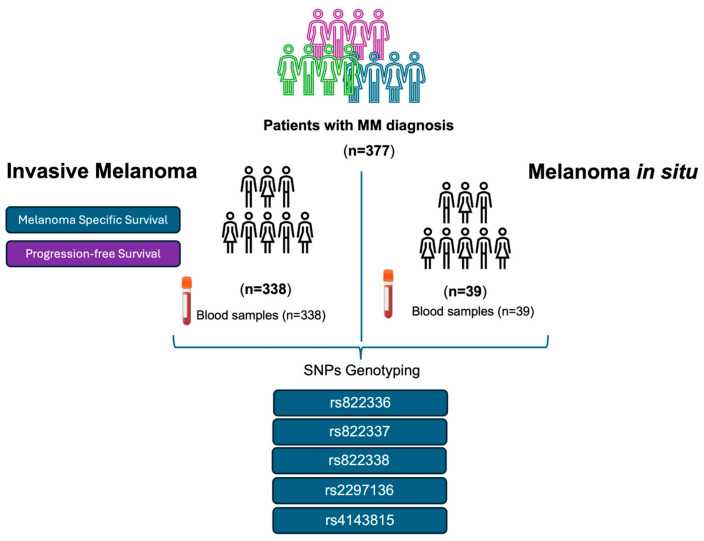
Patients with invasive melanoma and melanoma *in situ* were included in this study. Flowchart of the methodology carried out.

Following the systematic sampling method, we retrospectively screened blood samples from 377 patients of self-reported European origin with MM diagnosis who were followed by the Dermatology Department at “Complejo Hospitalario Universitario Nuestra Señora de Candelaria” (CHUNSC) (Santa Cruz de Tenerife, Spain) between the years 1975 and 2022 and who accepted to be included in this study. The samples corresponded to 338 patients with invasive melanoma and 39 with melanoma *in situ* (stage 0 of the AJCC TNM staging system). The following variables were analysed for the patients with invasive melanoma: sex, age at diagnosis, thickness (Breslow index), ulceration, and stage were analysed (Table 1). Figure 1 shows the patients included in this study and the workflow chart.

This study was approved by the local Ethics Committee (C.P. MO—C.I. PI-57/17 and C.P. MO—C.I. PI-39/14) of HUNSC. Informed consent was obtained from all participants.

### 2.2. Genotyping of PDCDL1 Single-Nucleotide Polymorphisms

DNA extractions from whole blood were performed using the ‘IllustraTM blood genomicPrep Mini Spin’ kit (GE Healthcare Life Science, Chicago, IL, USA). DNA concentration was measured using the NanoDrop ND-1000 spectrophotometer (Thermo Fisher Scientific Inc., Waltham, MA, USA). The study of *PDCDL1* gene variants consisted of the selection of five single-nucleotide polymorphisms (SNPs) previously reported of interest in the literature: rs822336, rs822337, rs822338, rs2297136, and rs4143815 [18,20,21,22,23]. The genotype analysis of the selected SNPs was carried out by Helix BioS Company (Madrid, Spain). The DNA samples were genotyped for the cited SNPs, in duplicate, with qPCR using TaqMan probes in a QuantStudio™ 12K Flex Real-Time PCR System (Thermo Fisher Scientific Inc., Waltham, MA, USA), giving a total of 4170 trials. Negative controls were also included in the assay. An average overall quality of 98.96 (+/−0.17)% was obtained in the SNP calling.

### 2.3. Statistical Analysis

Hardy–Weinberg equilibrium (HWE) was determined using the quick exact test for HWE by Wigginton, Cutler, and Abecasis from the SNPassoc package 1.9-2-1. Likewise, for each SNP, the frequency of the minor allele (MAF) for this study and the SNP calling rate (%) were estimated.

Different models of genetic inheritance were evaluated: co-dominant, dominant, and recessive. Initially, the genotypes for each SNP were coded under the co-dominant genetic model. The differences between the genotypes of the SNPs involved in patients with MM and their clinical characteristics were evaluated using contingency tables based on the Chi-square test corrected by Yeats in the case of qualitative variables. For age and Breslow index, the ANOVA test and the Kruskal–Wallis test were used, respectively, evaluating in pairs and correcting for multiple comparisons. The cut-off point for the significance level was set at 0.05, and bilateral contrasts were performed.

Melanoma-specific survival (MSS) and progression-free survival (PFS) were estimated using the Kaplan–Meier (KM) method. Differences between curves were evaluated using the log-rank test, analysing both clinical and genotypic variables. A Cox regression for proportional hazards was used to assess possible associations between clinical and genotypic characteristics and the MSS and PFS endpoints. The hazard ratio (HR) was estimated with its 95% confidence interval (95% CI) in a univariate and multivariate analysis. Significance levels were established at a *p* < 0.05 value. HR was represented by “forest plot” graphs.

Analysis was performed using R statistical software version 3.6.2 (http://www.r-project.org/, accessed on 30 October 2022). Survival analysis and graphs were carried out with the Survival 3.4-0 (https://CRAN.R-project.org/package=survival, accessed on 30 October 2022) and Survminer 0.4.9 software (https://CRAN.R-project.org/package=survminer, accessed on 30 October 2022). The forestploter 0.2.0 software (https://CRAN.R-project.org/package=forestploter, accessed on 30 October 2022) was used to create the forest plots and the treatment and analysis of the genetic variants with the SNPassoc 1.9-2-1 (https://CRAN.R-project.org/package=SNPassoc, accessed on 30 October 2022) and SNPStats v0.95 (https://www.snpstats.net/, accessed on 30 October 2022) software.

## 3. Results

Thirty-nine patients (10.3%) showed *in situ* MM. Their mean age at diagnosis was 51.0 (±18) years. Twenty-nine were female (74.4%). This group of patients was not included in the prognostic analysis, as they were cured after excision and appropriate widening of surgical margins. The mean age at diagnosis for invasive MM patients was 53.1 (±16.3) years, and 142 (42%) were men. Table 1 shows the main clinical features of the 338 cases with invasive MM by specific death and progression of MM.

### 3.1. Clinical Features and Survival Analyses

**Figure 2:**
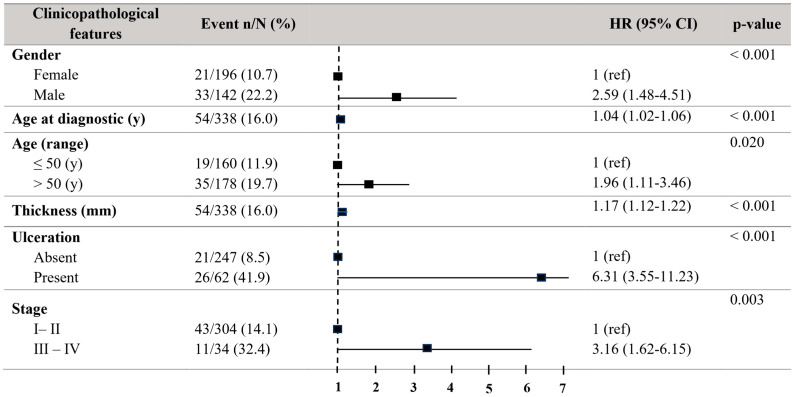
Forest plot with the clinicopathological characteristics of melanoma patients. Univariate analysis of melanoma-specific survival (MSS). Hazard ratio (HR) (CI 95%). Patients with HR > 1 present a high risk of death. Statistically significant *p*-values < 0.05.

Regarding melanoma-specific survival (MSS), patients older than 50 years showed a significant decrease in MSS (HR = 1.96, 95% CI = 1.11–3.46; *p* = 0.021) in the univariate Cox regression analyses. Males revealed a decreased MSS (HR = 2.59, 95% CI = 1.48–4.51; *p* < 0.001). The tumour thickness (HR = 1.17, 95% CI = 1.12–1.22; *p* < 0.001) and the presence of ulceration (HR = 6.31, 95% CI = 3.55–11.23; *p* < 0.001) were in relationship to decreased MSS. Stages III–IV exhibited a significantly reduced MSS (HR = 3.16, 95% CI = 1.62–6.15; *p* = 0.003) (Figure 2).

**Figure 3:**
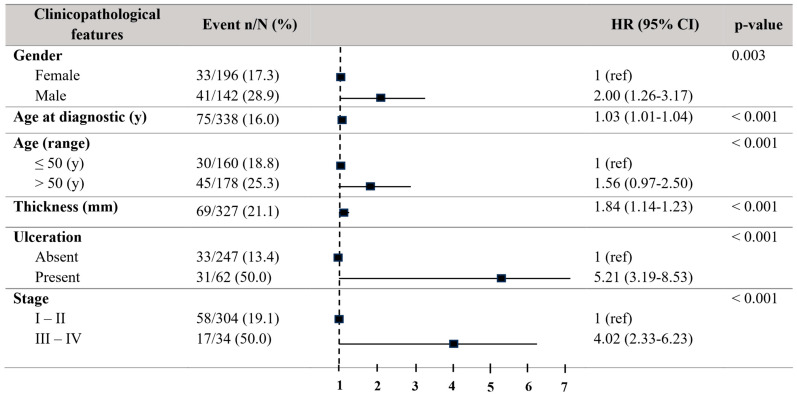
Forest plot with the clinicopathological characteristics of melanoma patients. Univariate analysis of progression-free survival (PFS). Hazard ratio (HR) (IC 95%). Patients with HR > 1 present a high risk of death. Statistically significant *p*-values < 0.05.

Concerning progression-free survival (PFS), patients older than 50 years also showed a decreased PFS (HR = 2.00, 95% CI = 1.26–3.17; *p* = 0.003). Males revealed a decreased PFS (HR = 2.00, 95% CI = 1.26–3.17; *p* = 0.003). The tumour thickness (HR01.84, 95% CI = 1.14–1.23; *p* < 0.001) and the existence of ulceration (HR = 5.21, 95% CI = 3.19–9.53; *p* < 0.001) disclosed a relationship to a reduced PFS. Stages III–IV also exhibited a significantly reduced PFS (HR = 4.02, 95% CI = 2.33–6.93; *p* < 0.001) (Figure 3).

### 3.2. PDCDL1 Polymorphisms and Survival Analyses

**Figure 4:**
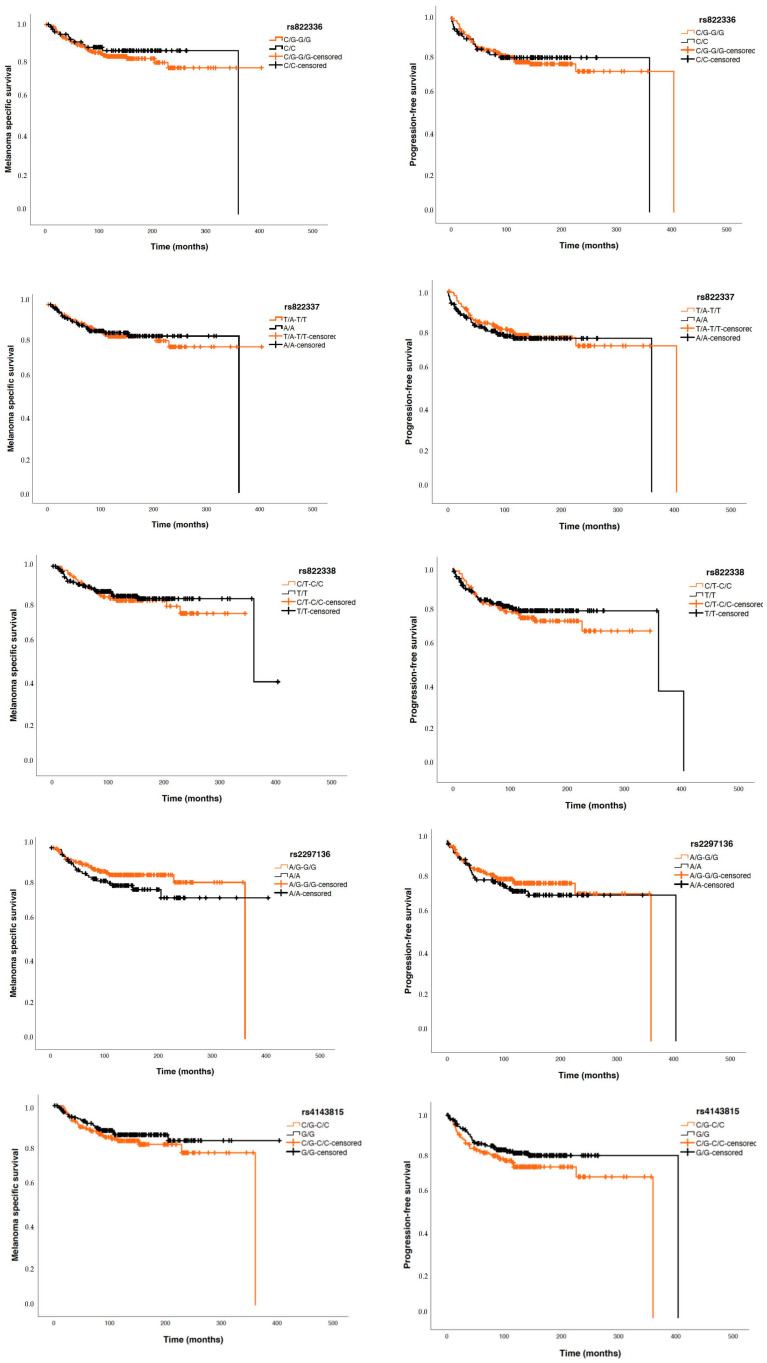
Kaplan–Meier curves for melanoma-specific survival (MSS) and progression-free survival (PFS) based on the five ana-lysed SNPs.

Considering patients with invasive tumours, Cox regression studies did not show significant differences between the five analysed *PDCDL1* polymorphisms and PFS nor MSS (Figure 4).

### 3.3. PDCDL1 Polymorphisms and Tumour Invasiveness

The genetic variants analysis revealed an association between four of the five studied *PDCDL1* SNPs and the invasiveness of the tumour. We found that patients who presented, under a dominant model of inheritance, the rs822336 CG or GG genotypes (OR = 3.01, 95% CI = 1.53–5.92; *p* = 0.0017), the TA or TT genotypes in rs822337 (OR = 2.45, 95% CI = 1.22–4.93; *p* = 0.0098), and the CT or CC genotype of rs822338 (OR = 2.23, 95% CI = 1.05–4.73; *p* = 0.028) were at an increased risk of developing invasive melanomas compared to those that presented an *in situ* melanoma (Table 2). On the other hand, those MM cases that harboured the AG or GG genotype in rs2297136 were at a lower risk of developing invasive tumours (OR = 0.29, 95% CI = 0.11–0.75; *p* = 0.0038) (Table 2).

The genetic analysis at the haplotype level resulted in similar findings. Patients harbouring the haplotype GTCAC, which contains the rs822336 rs822337, rs822338, and rs4143815 minor alleles and the rs2297136 frequent A allele, were at an increased risk of developing an invasive melanoma (OR = 2.95, 95% CI = 1.08–8.10, *p* = 0.036) (Table 3) compared to those who did not harbour it.

Regarding patients with invasive MM, a significant association was found between the SNP rs2297136 and the Breslow index. Patients carrying the homozygous AA genotype have thicker tumours (1.4 mm; IQR_25–75_: 0.6–2.6 mm) than those harbouring the AG (1.0 mm; IQR_25–75_: 0.6–2 mm) or GG (0.8 mm; IQR_25–75_: 0.5–1.7 mm) (*p* = 0.030). No association between other clinicopathological features and the studied gene variants was found. (Table 4).

## 4. Discussion

Melanoma is one of the most aggressive forms of skin cancer, and its development is influenced by various genetic factors. However, the exact contribution of genetic variants to melanoma susceptibility and clinical characteristics such as primary tumour thickness remains poorly understood. For example, relevant studies have found that the variants rs12913832 in HERC2, rs3798577 in ESR1 [25], and rs183471242 on chromosome 11 are linked to both MM risk and tumour thickness, suggesting that germline genetic variation plays a crucial role in disease development and progression [26]. Up to now, data regarding the relationship between *PDCDL1* SNPs and MM features have been scarce. This study provides novel descriptive and experimental analyses of the relationship between five *PDCDL1* single-nucleotide variants and relevant features of the disease. The activation of the PD-1/PD-L1 axis is vital for tumour cells to evade the immune system. This is why the study of genetic variants in genes involved in immune control may be used to predict the course of the disease and the response to cancer treatment [23,27].

To our knowledge, this is the first study that analyses *PDCDL1* gene variants and MM features. In our research, a significant association was found between four *PDCDL1* SNPs and the invasive features of MM. Patients with invasive MM exhibited higher frequencies of the G allele of rs822336, the T allele of rs822337, and the C allele of rs822338 compared to patients with *in situ* MM. These results suggest that carrying these alleles is associated with an increased risk of tumour invasiveness. Overlapping findings have been reported for the rs822336 and rs822337 polymorphisms in non-small-cell lung cancer: individuals carrying the G and T alleles, respectively, showed poorer overall survival [28].

The polymorphism rs22997136 has been the focus of previous research. The study by Boutros et al. (2023) investigated its role in predicting the tumour response and development of immune-related adverse effects in patients with advanced MM with anti-PD-1 treatment without finding any significant results [29]. In the current study, the patients carrying the G allele of rs2297136 showed a significant association with *in situ* MM. Consistent with this finding, in the group of cases with invasive MM, individuals carrying the homozygous genotype A/A in rs2297136 had significantly thicker tumours compared to those with the A/G or G/G genotypes. To our knowledge, the relationship between this SNP and tumour thickness has not been reported in previous studies. However, other gene variants such as rs12203592 in *IRF4* have already been associated with Breslow thickness and worse MSS [30] in patients of European origin.

The study of haplotypes, a combination of alleles of different loci on the same chromosome, has been valuable in identifying individuals at risk for developing a disease or its progression. An important finding in this study lies in the genetic association between the GTCAC haplotype (rs822336, rs822337, rs822338, rs2297136, rs4143815) in *PDCDL1* and the increased risk of developing invasive MM: those patients carry this haplotype at higher frequencies than patients with *in situ* melanomas. Few studies focus on the study of haplotypes in *PDCDL1*. The first one analysed an Iranian population with basal cell carcinoma (BCC) [31]. The second focused on rheumatoid arthritis in the Chinese population [32]. Given the scarcity of data on the subject, the findings established here are relevant for conducting further studies.

Addressing polymorphisms to predict the progression of diseases is challenging. Regarding cancer, results vary significantly based on the specific molecular profiles of each tumour, the tumour microenvironment, and the presence of genetic heterogeneity. Research suggests that the herein-studied gene variants may be related to the prognosis of other types of cancer. Harbouring the rs822336 CC genotype was reported as a favourable prognostic factor in gastric cancer in a Chinese population [33]. The SNPs rs822336 (G/C) and rs822337 (T/A) have been linked to worse survival in triple-negative breast cancer patients in a European population [20]. Additionally, rs4143815 C/G was reported to be associated with increased susceptibility to developing gastric cancer, bladder cancer, and hepatocellular carcinoma [18]. However, the current study did not observe significant associations between the analysed *PDCDL1* SNPs with MSS or PFS.

Importantly, genetic variants may influence the response to therapy. Therefore, the relationship between polymorphisms and disease prognosis is not always straightforward. For example, Gong et al. [34] found that Asian individuals with advanced non-small-cell lung cancer carrying the AA genotype of the rs2297136 SNP who received anti-PD-1 therapy had worse overall survival and PFS than those patients with the AG/GG genotype [34]. However, other investigations have provided contradictory results for the rs2297136 AA genotype and survival in ovarian [24] and gastric cancer [33]. These controversies may be due to differences in the tumour microenvironment, genetic regulation, and epigenetic modifications. Furthermore, population diversity is a crucial factor in the study of polymorphisms, as allele frequencies vary significantly across ethnic groups due to evolutionary history and local adaptations [35].

The rs2297136 is an A-to-G mutation in the 3′-UTR of *PDCDL1*. It is shown that rs2297136 could affect PD-L1 expression by modulating the miRNA–mRNA interaction [36,37]. An increase in the expression of PD-L1 in tumour cells or the tumour microenvironment, such as in tumour-associated macrophages, may allow melanoma to evade the immune response and facilitate an immunosuppressive environment that favours tumour growth, greater invasiveness and, therefore, a thicker tumour. The expression of PD-L1 is not necessarily associated with tumour development, as it may be induced as part of inflammatory processes that do not involve neoplasia or tumour development [38]. A meta-analysis including thirteen published articles with 1062 enrolled patients, conducted by Yang J. et al. [39], concluded that PD-L1 expression may not predict a worse prognosis in melanoma patients. Instead, elevated PD-L1 expression was found to be associated with the absence of lymph node metastasis [39].

As strengths of our study, we highlight the presence of a well-characterized cohort of patients with MM who underwent prolonged follow-up. The main clinical parameters were recorded for nearly every patient, and they are consistent with the data in the literature. Thus, it should be considered as a representative cohort [3,40]. Gender, Breslow index, ulceration, and stage at diagnosis were related to MSS and PFS [18,19,22]. However, our research also shows limitations. First, a larger series would provide more reliable results. Second, further genetic analyses adjusting for potential population structure would enhance the precision of our findings. Lastly, we recognise the importance of confirming the expression of PD-L1 in the tumour tissue. This data would allow us to complement the present findings, so further research will focus on it.

## 5. Conclusions

This study supports that SNPs in the *PDCDL1* gene are associated with invasive features of MM. In particular, the G allele of rs822336, the T allele of rs822337, and the C allele of rs822338 are associated with a higher risk of tumour invasiveness. In addition, the A/A genotype of rs2297136 is associated with thicker tumours. No relationship with MSS or PFS was found. Further studies on *PDCDL1* polymorphisms, considering diverse genetic and epigenetic factors, are necessary to understand their impact on cancer prognosis and treatment. Further large-scale epidemiologic studies are needed to confirm present findings.

## Figures and Tables

**Table 1:** Clinicopathological characteristics of participating MM patients vs. melanoma progression and death due to melanoma.

			Death Due to Melanoma (n [%])		Melanoma Progression (n [%])	
**Clinical characteristics**	N	Patients (n [%])	No	Yes	No	Yes
**Age at diagnosis (years) ^1^**	338	53.1 (16.3)	51.8 (15.7)	60.1 (17.7)	51.8 (15.7)	57.7 (17.9)
**Age at diagnosis**	338					
≤50 years		160 (47.3)	141 (88.1)	19 (11.9)	130 (81.3)	30 (18.8)
>50 years		178 (52.7)	143 (80.3)	35 (19.7)	133 (74.7)	45 (25.3)
**Gender**	338					
Female		196 (58.0)	175 (89.3)	21 (10.7)	162 (82.7)	34 (17.4)
Male		142 (42.0)	109 (76.8)	33 (23.2)	101 (71.1)	41 (28.9)
**Breslow (mm) ^2^**	338	1.10 (0.60–2.15)	0.86 (0.57–1.75)	3.10 (1.90–5.00)	0.80 (0.55–1.70)	2.50 (1.60–5.00)
**Ulceration**	309					
Absent		247 (79.9)	226 (91.5)	21 (8.5)	214 (86.6)	33 (11.3)
Present		62 (20.1)	36 (58.1)	26 (41.9)	31 (50.0)	31 (50.0)
**Stage**	338					
I–II		304 (89.9)	261 (85.9)	43 (14.1)	246 (80.9)	58 (19.1)
III–IV		34 (10.1)	23 (67.7)	11 (32.4)	17 (50.0)	17 (50.0)

^1^ Mean and standard deviation of the age (mean [DE]). ^2^ Median and interquartile range of the Breslow (median (IQR_25–75_)).

**Table 2:** Association of Single-Nucleotide Polymorphisms (SNPs) in *PDCDL1* with tumour invasion.

*PDCDL1* SNPs	*In Situ* Melanoma	Invasive Melanoma	OR (95% CI)	*p*-Value
	n (%)	n (%)		
**rs822336**				
Codominance				0.0058 **
CC	19 (48.7%)	81 (24%)	1.00	
CG	15 (38.5%)	175 (51.8%)	2.74 (1.32–5.66)	
GG	5 (12.8%)	82 (24.3%)	3.85 (1.37–10.80)	
Dominance				0.0017 **
CC	19 (48.7%)	81 (24%)	1.00	
CG + GG	20 (51.3%)	257 (76%)	3.01 (1.53–5.92)	
Recessive				0.089
CC + CG	34 (87.2%)	256 (75.7%)	1.00	
GG	5 (12.8%)	82 (24.3%)	2.18 (0.82–5.75)	
**rs822337**				
Codominance				0.03 *
AA	26 (66.7%)	152 (45%)	1.00	
TA	10 (25.6%)	155 (45.9%)	2.65 (1.24–5.69)	
TT	3 (7.7%)	31 (9.2%)	1.77 (0.50–6.21)	
Dominance				0.0098 **
AA	26 (66.7%)	152 (45%)	1.00	
TA + TT	13 (33.3%)	186 (55%)	2.45 (1.22–4.93)	
Recessive				0.76
AA + TA	36 (92.3%)	307 (90.8%)	1.00	
TT	3 (7.7%)	31 (9.2%)	1.21 (0.35–4.16)	
**rs822338**				
Codominance				0.015 *
TT	29 (74.4%)	191 (56.5%)	1.00	
CT	7 (17.9%)	135 (39.9%)	2.93 (1.25–6.88)	
CC	3 (7.7%)	12 (3.5%)	0.61 (0.16–2.28)	
Dominance				0.028 *
TT	29 (74.4%)	191 (56.5%)	1.00	
CT + CC	10 (25.6%)	147 (43.5%)	2.23 (1.05–4.73)	
Recessive				0.26
TT + CT	36 (92.3%)	326 (96.5%)	1.00	
CC	3 (7.7%)	12 (3.5%)	0.44 (0.12–1.64)	
**rs2297136**				
Codominance				0.0097 **
AA	5 (12.8%)	115 (34%)	1.00	
AG	21 (53.9%)	156 (46.1%)	0.32 (0.12–0.88)	
GG	13 (33.3%)	67 (19.8%)	0.22 (0.08–0.66)	
Dominance				0.0038 **
AA	5 (12.8%)	115 (34%)	1.00	
AG + GG	34 (87.2%)	223 (66%)	0.29 (0.11–0.75)	
Recessive				0.063
AA + AG	26 (66.7%)	271 (80.2%)	1.00	
GG	13 (33.3%)	67 (19.8%)	0.49 (0.24–1.01)	
**rs4143815**				
Codominance				0.38
GG	23 (59%)	187 (55.3%)	1.00	
CG	15 (38.5%)	124 (36.7%)	1.02 (0.51–2.03)	
CC	1 (2.6%)	27 (8%)	3.32 (0.43–25.57)	
Dominance				0.66
GG	23 (59%)	187 (55.3%)	1.00	
CG + CC	16 (41%)	151 (44.7%)	1.16 (0.59–2.28)	
Recessive				0.17
GG + CG	38 (97.4%)	311 (92%)	1.00	
CC	1 (2.6%)	27 (8%)	3.30 (0.44–24.95)	

A: adenine; T: thymine; C: cytosine; G: guanine. OR: Odds ratio, CI: confidence interval. * *p*-value: <0.05; ** *p*-value: <0.01.

**Table 3:** Distribution of the *PDCDL1* haplotypes and their association with tumour invasiveness (*in situ* vs. invasive).

rs822336	rs822337	rs822338	rs2297136	rs4143815	Total	*In Situ* MM	Invasive MM	OR (95% CI)	*p*-Value
C	A	T	G	G	0.3273	0.487	0.3104	1.00	-
C	A	T	A	G	0.1415	0.1477	0.1415	1.39 (0.66–2.94)	0.39
**G**	**T**	**C**	**A**	**C**	0.1104	0.0621	0.1157	2.95 (1.08–8.10)	0.036 *
G	A	T	A	C	0.0848	0.0601	0.0893	2.22 (0.78–6.38)	0.14
G	A	T	A	G	0.0778	0.0553	0.08	2.14 (0.71–6.42)	0.18
G	T	C	G	G	0.0609	0.0786	0.057	1.01 (0.42–2.46)	0.98

A: adenine; T: thymine; C: cytosine; G: guanine. OR: Odds ratio, CI: confidence interval. In bold is the haplotype GTCAC. * *p*-value: <0.05.

**Table 4:** Association analysis between the five polymorphisms of the *PDCDL1* gene studied and the main clinical characteristics of patients with melanoma.

	rs822336 (n [%])				rs822337 (n [%])				rs822338 (n [%])				rs2297136 (n [%])				rs4143815 (n [%])			
Clinical Characteristics	CC	CG	GG	*p*	AA	TA	TT	*p*	TT	CT	CC	*p*	AA	AG	GG	*p*	GG	CG	CC	*p*
**Age at diagnosis (years) ¹**	53.5 (14.8)	53.6 (16.9)	52.0 (16.5)	0.748	54.0 (16.2)	52.1 (16.0)	54.5 (18.0)	0.528	53.3 (16.2)	52.8 (16.2)	54.8 (18.2)	0.902	52.8 (16.0)	53.1 (16.9)	53.9 (15.5)	0.916	53.8 (15.4)	52.0 (17.6)	53.8 (15.6)	0.629
**Age at diagnosis**																				
**≤50 years**	42 (51.9)	75 (42.9)	43 (52.4)	0.231	72 (47.4)	73 (47.1)	15 (48.4)	0.991	91 (47.6)	63 (46.7)	6 (50.0)	0.968	55 (47.8)	74 (47.4)	31 (46.3)	0.979	88 (47.1)	62 (50.0)	10 (37.0)	0.471
**>50 years**	39 (48.1)	100 (57.1)	39 (47.6)		80 (52.6)	82 (52.9)	16 (51.6)		100 (52.4)	72 (53.3)	6 (50.0)		60 (52.2)	82 (52.6)	36 (53.7)		99 (52.9)	62 (50.0)	17 (63.0)	
**Gender**																				
**Female**	49 (60.5)	99 (56.6)	48 (58.5)	0.834	85 (55.9)	94 (60.6)	17 (54.8)	0.656	107 (56.0)	80 (59.3)	9 (75.0)	0.403	68 (59.1)	91 (58.3)	37 (55.2)	0.870	109 (58.3)	72 (58.1)	15 (55.6)	0.964
**Male**	32 (39.5)	76 (43.4)	34 (41.5)		67 (44.1)	61 (39.4)	14 (45.2)		84 (44.0)	55 (40.7)	3 (25.0)		47 (40.9)	65 (41.7)	30 (44.8)		78 (41.7)	52 (41.9)	12 (44.4)	
**Breslow (mm) (IQR) ^2^**	0.9 (0.6–2.0)	1.1 (0.6–2.4)	1.3 (0.7–2.1)	0.574	0.9 (0.6–2.5)	1.2 (0.6–2.2)	1.2 (0.8–1.9)	0.607	0.9 (0.6–2.4)	1.2 (0.6–2.1)	1.2 (0.4–1.9)	0.576	1.4 (0.6–2.6)	1.0 (0.6–2.0)	0.8 (0.5–1.7)	0.030 *	1.0 (0.6–2.1)	1.1 (0.6–2.1)	1.2 (0.7–2.2)	0.701
**Ulceration**																				
**Absent**	61 (81.3)	124 (78.5)	62 (81.6)	0.807	108 (78.8)	113 (79.0)	26 (89.7)	0.389	142 (81.1)	96 (78.0)	9 (81.8)	0.796	75 (73.5)	121 (83.4)	51 (82.3)	0.140	141 (82.0)	87 (77.7)	19 (76.0)	0.593
**Presence**	14 (18.7)	34 (21.5)	14 (18.4)		29 (21.2)	30 (21.0)	3 (10.3)		33 (18.9)	27 (22.0)	2 (18.2)		27 (26.5)	24 (16.6)	11 (17.7)		31 (18.0)	25 (22.3)	6 (24.0)	
**Stage**																				
**I** **–** **I** **I**	70 (86.4)	162 (92.6)	72 (87.8)	0.239	134 (88.2)	143 (92.3)	27 (87.1)	0.421	170 (89.0)	124 (91.9)	10 (83.3)	0.520	98 (85.2)	143 (91.7)	63 (94.0)	0.101	169 (90.4)	111 (89.5)	24 (88.9)	0.953
**III** **–** **IV**	11 (13.6)	13 (7.4)	10 (12.2)		18 (11.8)	12 (7.7)	4 (12.9)		21 (11.0)	11 (8.1)	2 (16.7)		17 (14.8)	13 (8.3)	4 (6.0)		18 (9.6)	13 (10.5)	3 (11.1)	

^1^ Age (years), mean and standard deviation (mean (SD)). ^2^ Median Breslow (mm) and (IQR) interquartile range (median (IQR_25–75_%)); * *p* < 0.05.

## Data Availability

Data are available upon reasonable request. All data relevant to this study are included in the article.
